# Timing and Outcomes of Intracranial Stenting in the Post-SAMMPRIS Era: A Systematic Review

**DOI:** 10.3389/fneur.2021.637632

**Published:** 2021-02-04

**Authors:** Yanying Yu, Tao Wang, Kun Yang, Xiao Zhang, Simon Chun Ho Yu, Jichang Luo, Bin Yang, Yabing Wang, Yan Ma, Peng Gao, Liqun Jiao

**Affiliations:** ^1^Department of Neurosurgery, Xuanwu Hospital, Capital Medical University, Beijing, China; ^2^School of Medicine, Peking Union Medical College, Beijing, China; ^3^Department of Evidence-Based Medicine, Xuanwu Hospital, Capital Medical University, Beijing, China; ^4^Department of Imaging and Interventional Radiology, Prince of Wales Hospital, The Chinese University of Hong Kong, Shatin, China; ^5^Department of Interventional Radiology, Xuanwu Hospital, Capital Medical University, Beijing, China

**Keywords:** intracranial stenting, timing, systematic review, safety, efficacy

## Abstract

**Objective:** To investigate the impact of timing on the safety and efficacy of stenting for ICAS, we reviewed high-volume randomized controlled trials or prospective cohort studies of stenting for intracranial atherosclerotic artery stenosis (ICAS) after the SAMMPRIS trial.

**Methods:** We included randomized controlled trials or prospective cohort studies since 2011 (the publication of the SAMMPRIS trial), evaluating the outcomes of intracranial stenting for ICAS patients. The primary outcomes were perioperative and 1-year stroke or death rate. The interaction of timing and outcomes were shown on trend plots. Overall meta-analysis and subgroup analysis by timing of intracranial stenting were conducted.

**Results:** Fourteen studies with a total of 1,950 patients were included. The perioperative and post-operative stroke or death rates decreased with the time of stenting to the qualifying events. The perioperative stroke rate was significantly higher in patients treated within 21 days after the qualifying events, compared to those beyond 21 days (IRR = 1.60, 95%CI: 1.10–2.33; *p* = 0.014), similar relationships were obtained for both post-procedural (IRR = 1.61, 95%CI: 1.02–2.55; *p* = 0.042) and 1-year (IRR = 1.51, 95%CI: 1.10–2.08; *p* = 0.012) stroke or death rate.

**Conclusions:** The timing of intracranial stenting may influence the safety and efficacy outcomes of stenting. Intracranial stenting within 21 days from the qualifying events may confer a higher risk of stroke or death. More studies are needed to confirm the impact of timing and the proper cut-off value.

## Introduction

Intracranial atherosclerosis artery stenosis (ICAS) is one of the major causes of ischemic stroke. Reversal of the stenosis with intracranial stenting had shown the potential to reduce the risk of recurrent stroke significantly, while its safety and efficacy has remained controversial since the publication of the SAMMPRIS trial in 2011, in which the 30-day stroke or death rate in the stenting arm were higher compared with aggressive medical management (1). Based on SAMMPRIS and VISSIT trial, medical management rather than stenting is recommended with symptomatic ICAS, while stenting is approved for patients who had ≥2 strokes despite medical treatment (2, 3). However, with the proper patient selection and the best interventional techniques being revealed gradually, the intracranial stenting shows more promising efficacy, still being expected to improve the treatment of ICAS. In the latest WEAVE study (Wingspan Stent System Post Market Surveillance), the timing of intracranial stenting has been suggested as an essential factor relating to the outcomes (4). Early stenting after the qualifying event seemed to be associated with a higher risk of post-operative complications (5). However, the specific relationship between timing of intracranial stenting and short- and long-term outcomes in ICAS patients was still unclear. Therefore, this systematic review aimed to investigate the impact of timing on the safety and efficacy outcomes of stenting for ICAS and propose a possible timing cut-off.

## Method

The study is reported following the Preferred Reporting Items for Systematic Reviews and Meta-Analyses (PRISMA) statement (6). We searched PubMed, the Cochrane Library and EMBASE for RCT or prospective studies reporting ≥50 cases of stenting for ICAS since 2011 (the publication of the SAMMPRIS trial) (Supplementary Table 1) to March 1st, 2020. Two researchers individually screened the titles and abstracts, and further assessed the potential studies after viewing the full text. Studies reporting patients with symptomatic ICAS (≥50%) who underwent intracranial stenting (including self-expanding stent and balloon-mounted stent) without angioplasty were included. The eligibility criteria presented in the PICOS pattern were shown in Supplementary Table 2. The risks of bias were assessed with the Cochrane Collaboration's tool for RCT and the adapted Newcastle-Ottawa Scale for observational studies (7).

The correlation between the timing of the stenting and the outcomes were analyzed using the Pearson Correlation Coefficient and described in the trend graph. The studies were classified into timing ≤ 21 days group and timing >21 days group. Overall summaries of the meta-estimates (with 95% confidence intervals) were reported. Heterogeneity across studies and in subgroups was evaluated with the *I*^2^ statistic (*I*^2^ ≥ 70% was considered as high heterogeneity), and fixed or random effect models were used accordingly. Poisson regression was used to analyze the pooled outcomes. The incidence rate ratio (IRR) between subgroups was calculated to compare the outcomes in the two groups. A sensitivity analysis was performed to explore possible explanations for heterogeneity, and three studies with much longer intervals of stenting were excluded.

### Data Availability

Our study is based on published studies and data only.

## Results

Two RCTs and 12 prospective observational studies including a total of 1,950 patients with symptomatic, severe (70–99%) ICAS were finally included from 1,981 references. The timing of stenting in these studies ranged from 7 to 132.9 days. The assessment of bias showed a low risk (Supplementary Table 3, Supplementary Figures 1–4).

**Figure 1:**
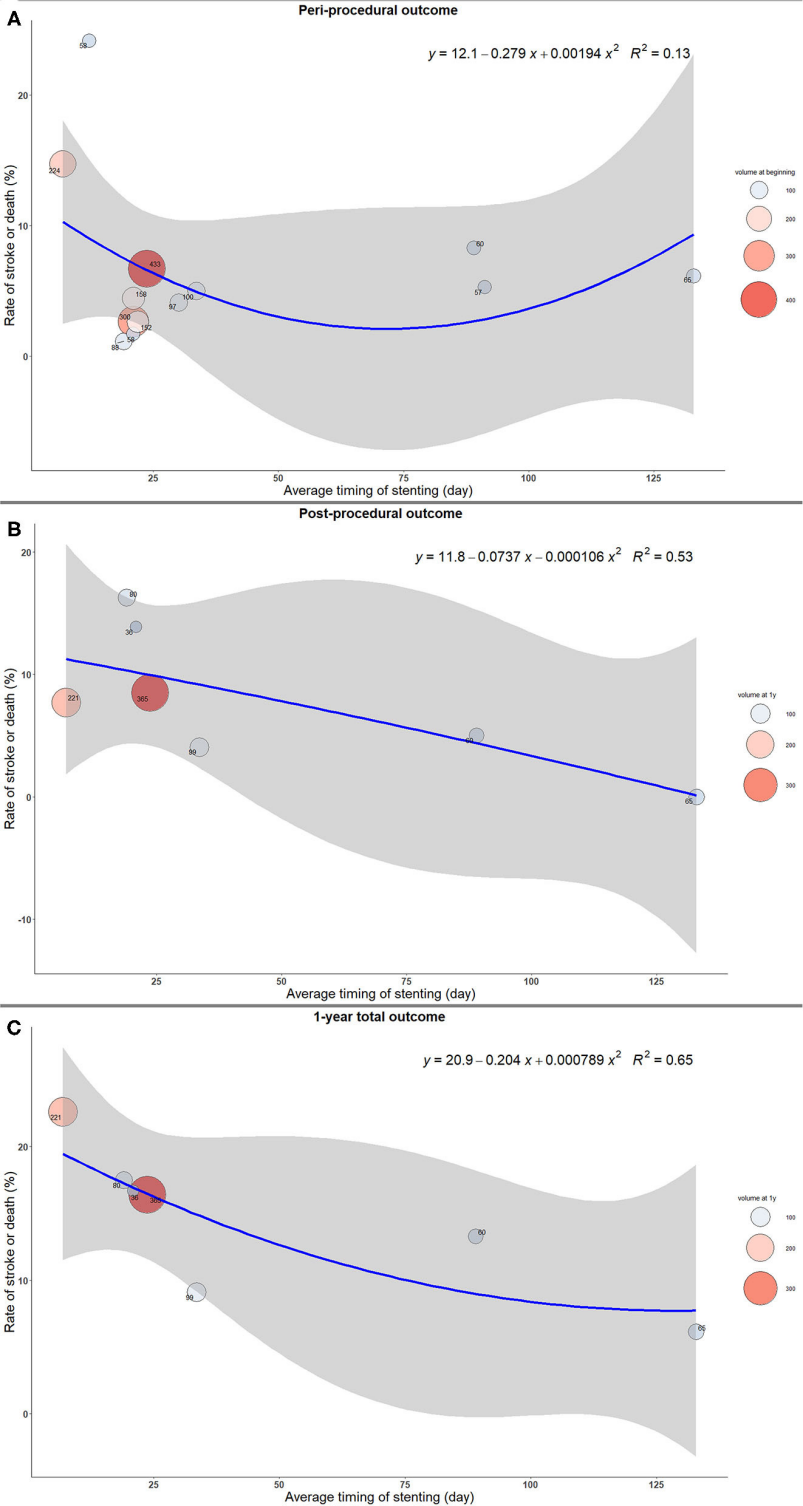
The trend of rate of stroke or death with timing of intracranial stenting. **(A)** Peri-procedural outcome. **(B)** Post-procedural outcome. **(C)** 1-year total outcome.

**Figure 2:**
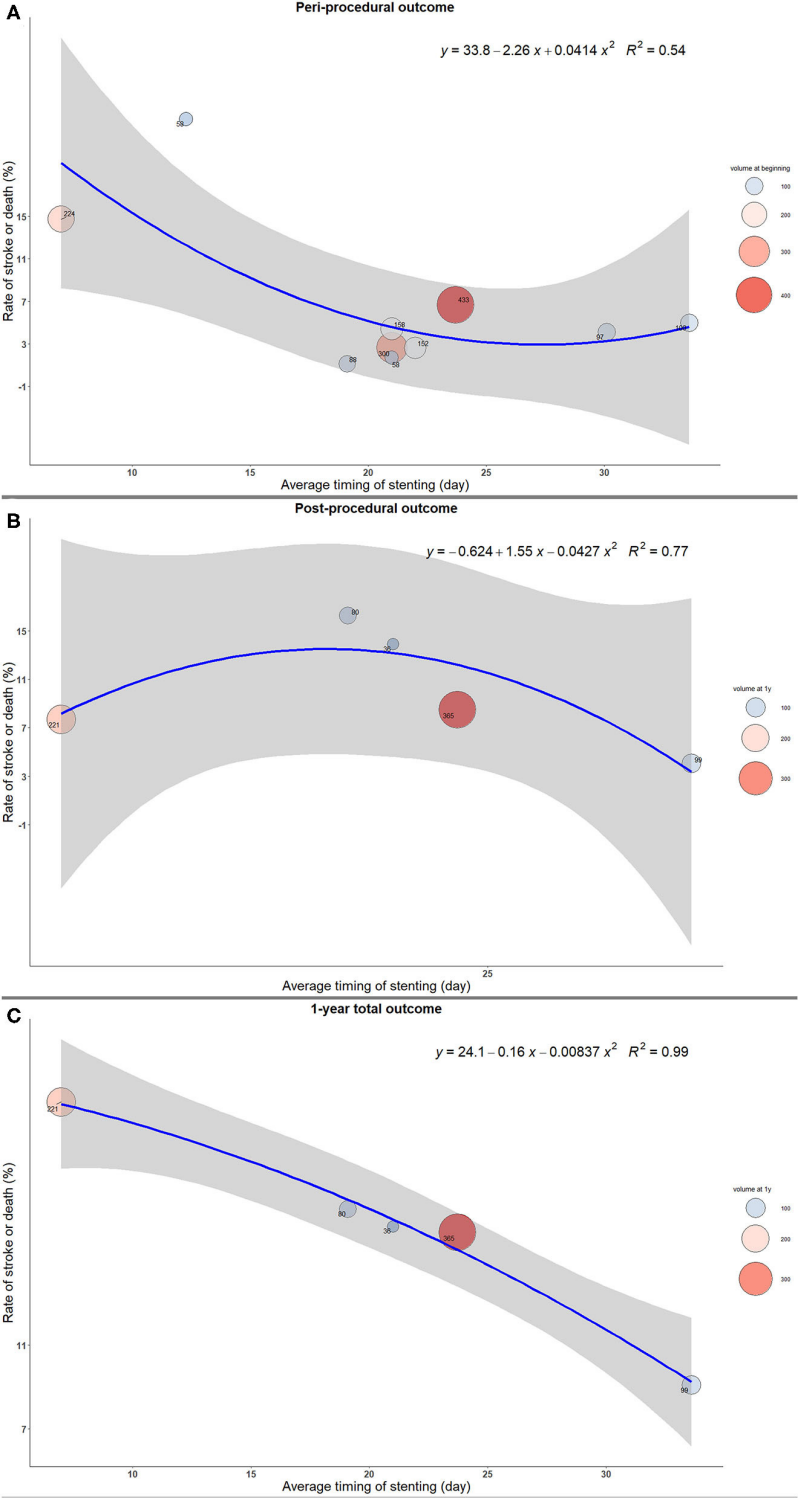
The trend of rate of stroke or death with timing of intracranial stenting in sensitivity analysis. **(A)** Peri-procedural outcome. **(B)** Post-procedural outcome. **(C)** 1-year total outcome.

The stroke or death rates were the highest when the stenting was implemented early from the qualifying event in both peri- and post-procedural period, as well as at 1 year after the procedure (Figure 1). Similar results were shown in the sensitivity analysis after excluding three studies with significantly delayed timing of stenting. The complication rates decreased as the interval increased to 20 days, and the changes became mild afterwards (Figure 2).

The pooled estimate of 1-year total stroke or death was 20.55% (95%CI: 16.33–25.11%) in the timing ≤ 21 days group, which was significantly higher than that in the timing >21 days group (11.62%; 95%CI: 7.16–16.94%; *p* = 0.012), with low or moderate heterogeneity (Table 1 and Supplementary Table 7). In the meta-regression, the perioperative stroke rate was significantly higher in patients treated within 21 days after the qualifying events, compared to those beyond 21 days (IRR 1.60, 95%CI: 1.10–2.33; *p* = 0.014), similar relationships were obtained for both post-procedural (IRR = 1.61, 95%CI: 1.02–2.55; *p* = 0.042) and 1-year (IRR = 1.51, 95%CI: 1.10–2.08; *p* = 0.012) stroke or death rate (Table 1).

**Table 1:** Pooled analysis and meta-regression analysis of outcomes of intracranial stenting between time interval of ≤ 21 and >21 days.

**Outcomes**	**No. of studies**	**Total (%) (95% CI)**	**Timing ≤ 21 days (%) (95% CI)**	**Timing > 21 days (%) (95%CI)**	**$I_{T}^{2}$ (%)**	**$I_{\leq }^{2}$ (%)**	**$I_{>}^{2}$ (%)**	**IRR (95% CI)**	***P*-Value**
**Peri-procedural outcomes**									
Stroke	14	4.76 (2.67–7.35)	6.33 (1.82–13.02)	3.83 (2.35–5.60)	79.94	90.35	32.5	1.60 (1.10–2.33)	**0.014**
Death	14	0.68 (0.10–1.60)	0.42 (0.00–1.94)	0.93 (0.09–2.31)	60.7	69.08	55.3	0.74 (0.31–1.78)	0.501
Stroke or death	14	5.38 (3.23–7.99)	6.33 (1.82–13.02)	4.83 (3.44–6.43)	78.44	90.35	10.9	1.37 (0.96–1.96)	0.084
**Post-procedural outcomes**									
Stroke	8	5.98 (3.55–8.91)	8.24 (5.61–11.27)	3.59 (0.98–7.47)	58.75	0.00	66.77	1.64 (1.01–2.66)	**0.048**
Death	8	1.98 (0.50–4.15)	3.92 (0.45–9.79)	1.06 (0.08–2.72)	64.92	75.84	32.84	2.17 (1.01–4.67)	**0.048**
Stroke or death	7	6.69 (3.46–10.78)	11.47 (5.76–18.88)	3.91 (0.78–8.88)	74.35	60.69	77.21	1.61 (1.02–2.55)	**0.042**
**1-year total outcomes**									
Stroke	8	14.58 (9.87–19.97)	20.40 (12.37–29.77)	10.79 (7.57–14.47)	76.1	72.99	29.73	1.77 (1.29–2.43)	**0.000**
Death	8	4.32 (2.45–6.61)	6.13 (2.58–10.85)	3.06 (1.57–4.94)	48.02	54.61	11.11	1.79 (0.99–3.24)	0.055
Stroke or death	7	14.62 (10.58–19.16)	20.55 (16.33–25.11)	11.62 (7.16–16.94)	63.79	0.00	60.02	1.51 (1.10–2.08)	**0.012**

*CI confidence interval, I^2^ the variation attributable to heterogeneity, $I_{T}^{2}$ heterogeneity in overall meta-analysis, $I_{\leq }^{2}$ heterogeneity in the timing ≤ 21 days group, $I_{>}^{2}$ heterogeneity in the timing > 21 days group, IRR incidence rate ratio, univariate meta-regression analysis was adjusted by time of intervention (timing ≤ 21days compared to timing >21 days). The bold values represent the results with statistical significance (p < 0.05)*.

## Discussion

In this systematic review with a total sample size of 1,950, we assessed the effect of timing on outcomes of intracranial stenting in patients with symptomatic severe ICAS. The results showed (1) timing may influence both short- and long-term outcomes of stenting; (2) early stenting after the qualifying event may be associated with a higher risk of complication, while the difference is less obvious when the interval spans long.

Early stenting after the qualifying event predominantly leads to higher peri-procedural risks due to plaque detachment and reperfusion hemorrhage (8, 9). Previous studies of intracranial stenting, and those involving carotid artery stenting and intra-extracranial bypass surgery also had similar conclusions (10, 11). During the acute phase, the stent and the stenting procedure increase the fragility of the plaque and lead to disorders of blood-brain barrier. In contrast, a waiting period after the ischemic event allows cerebrovascular self-regulation and stabilization of plaque, thus avoiding some of the above adverse effects (5, 9).

In this study, the possible impact of timing of stenting on long-term outcomes was also shown, as all post-procedural complication rates were higher in the timing ≤ 21 days group. The stabilization of the plaques during the waiting period may decrease the risk of its rupture, increasing the efficacy of intracranial stenting and reducing the risk of restenosis (5, 12). In addition to the cerebrovascular self-regulation, delayed stenting may also enable the patients a better medical preparation for the procedure.

The proper cut-off point of timing is yet to be determined. Previous studies with a relatively lower level of evidence (observational single center studies) suggested 14 days as the cut-off point, (5) and some repocrted stenting in <10 or 18 days as risk factors for complications (12, 13). In this study, it is found that a time interval of 3 weeks (21 days) from the qualifying event to intracranial stenting may be an optimal cut-off point. In the pairwise comparison of the present study, both the short- and long-term outcomes were significantly different between the time intervals. No additional effect was seen when the deferred stenting was performed. This time interval has now been commonly accepted in Asian intracranial stenting trials and resulted in a low risk of complications in multiple studies (14).

Our study has some limitations. Firstly, limited to the number of the studies included, meta-regression analysis on the other risk factors (gender, age, percentage of stenosis, etc.) were not performed. The heterogeneity of the studies cannot be ignored. More researches are needed to address the association between other risk factors and the outcomes. Secondly, the timing of stenting was analyzed as the mean/median time interval as reported in the included studies. As the timing might be quite dispersed as individuals, the bias cannot be ignored. In addition, observational studies, though prospective studies, had inherited risk of bias. Random controlled trials with a large scale are needed in the future.

## Conclusion

The timing of intracranial stenting has an impact on its safety and efficacy outcomes. Intracranial stenting within 21 days from the qualifying events may confer a higher risk of stroke or death. More studies are needed to confirm the impact of timing and the optimal cut-off value.

## Data Availability Statement

The original contributions presented in the study are included in the article/Supplementary Material, further inquiries can be directed to the corresponding author/s.

## Ethics Statement

Ethical review and approval was not required for the study on human participants in accordance with the local legislation and institutional requirements. Written informed consent for participation was not required for this study in accordance with the national legislation and the institutional requirements. Written informed consent was obtained from the individual(s) for the publication of any potentially identifiable images or data included in this article.

## Author Contributions

YY and TW contributed to the study concept and design, KY and YY contributed to the analysis and interpretation of data. XZ and YY contributed to the drafting of the manuscript. SY, JL, BY, and YW contributed to the analysis and interpretation of data. YM, PG, and LJ contributed to the critical revision of the manuscript. All authors contributed to the article and approved the submitted version.

## Conflict of Interest

The authors declare that the research was conducted in the absence of any commercial or financial relationships that could be construed as a potential conflict of interest.
