# Supplementary material for: Timing and Outcomes of Intracranial Stenting in the Post-SAMMPRIS Era: A Systematic Review
Source: Front Neurol. 2021 Feb 4;12:637632. doi: 10.3389/fneur.2021.637632 (PMC7890236; doi:10.3389/fneur.2021.637632)
Supplement: Supplementary file 1 [file Table_1.DOCX]

**Supplementary file for “****Timing and outcomes of intracranial stenting in the post-SAMMPPRIS era: a systematic review”**

**Method**

**The manuscript was prepared in accordance with Preferred Reporting Items for Systematic Reviews and Meta-Analyses (PRISMA) statement.**

**Search strategy**

Literature search was performed by two independent reviewers (TW and YY) using PubMed. We The reference lists of selected studies and published review articles for other potential studies were also scrutinized. Other databases such as the ISRCTN registry, government registries and WHO registries were also searched for ongoing and recently completed studies. When necessary, the authors or conductors of the studies were consulted for further information.

**Table e-1.** PubMed search strategy.

| **Search step** | **Search terms** |
| --- | --- |
| #1 | intracranial atherosclerosis [MeSH] OR intracranial arterial stenosis [All Fields] OR intracranial atherosclerosis [All Fields] OR intracranial stenosis [All Fields] OR intracranial vertebral artery stenosis [All Fields] OR intracranial atherosclerotic stenosis [All Fields] OR intracranial vertebrobasilar artery stenosis [All Fields] OR ischemia cerebrovascular disease caused by artery stenosis [All Fields] OR basilar artery stenosis [All Fields] OR cerebral artery stenosis [All Fields] OR vertebral artery stenosis [All Fields] OR intracranial atherosclerotic diseases [All Fields] OR Atherosclerotic Vertebrobasilar Artery Occlusion [All Fields] OR intracranial large artery stenoses and occlusions [All Fields] OR intracranial vertebral artery atherosclerotic stenosis [All Fields] OR vertebral atherosclerotic diseases [All Fields] OR intracranial internal carotid artery stenosis [All Fields] OR atherosclerotic intracranial stenosis[All Fields] |
| #2 | angioplasty [MeSH] OR stenting [All Fields] OR angioplasty [All Fields] OR intracranial stent [All Fields] OR endovascular therapy [All Fields] OR Balloon-Expandable Intracranial Stenting [All Fields] OR Percutaneous transluminal angioplasty and stenting [All Fields] OR Endovascular treatment [All Fields] OR balloon-expandable intracranial stent [All Fields] |
| #3 | #1 AND #2 |
| #4 | Filters: Publication date from 2011/01/01; English |
| #5 | #3 AND #4 |

**Selection of studies**

Two reviewers (XW and YY) independently screened all the results, and selected the studies fulfilling all the criteria in Supplementary Table 2. Before screening and selection, a pilot trial was conducted to ensure the accordance on the criteria for inclusion and exclusion. Reviewers firstly excluded irrelevant studies by screening the titles, key words and abstracts, then acquired the full articles of all the remaining studies. Subsequently, reviewers read through the studies to assess eligibility for inclusion. In this step, the reasons of all excluded studies were recorded. When more than one articles were based on data from the same trial, we selected the latest study or the one with largest sample size. When disagreement arose, it was settled by consensus or a third reviewer (TW).

**Table e-2.** Eligibility criteria

| Patients: | Patients with symptomatic intracranial atherosclerotic arterial stenosis (≥ 50%) |
| --- | --- |
| Intervention: | Intracranial stenting (including self-expanding stent and balloon-mounted stent) without angioplasty. The time interval between qualifying event and stent placement was reported. |
| Comparison: | Compared with medical treatment or with no comparison. |
| Outcome: | The all reason death or stroke rate 1) during perioperative period or 30 days after the stenting; 2) during post-procedural period or 2 months to 1 year after the stenting; 3) at 1year after the stenting |
| Study type: | Randomized controlled trail or prospective cohort studies, including at least 50 patients, published in or after 2011 |

**Figure e-1** Study flow

**Risk of bias assessment**

Risk of bias was assessed independently by two reviewers (YY and KW) in each included study. The risk of bias was assessed based on Cochrane risk of bias tool for RCT and the method described in Methodological Quality and Synthesis of Case Series and Case Reports. We evaluated the bias of each study with regard to four domains: selection, ascertainment; causality and reporting.

**Figure e-2.** Risk of bias graph for non-RCT studies

**Figure e-3.** Risk of bias graph for RCT studies

**Figure e-4.** Risk of bias summary for non-RCT studies

**Figure e-5.** Risk of bias summary for RCT studies

Data extraction

The following data were extracted: (1) study characteristics; (2) patient: stenosis degree, lesion location;(3) mean/median time interval between qualifying event to stent placement; stent type; (4) stroke or death rate at peri-procedure, post-procedure and at 1 year as outcomes.

**Table e-3.** Detailed characteristics of the included studies.

| Study ID | Publication time | Timing(d) | Country | Sample size | Mean age, year | Sex/male, % | Preprocedural stenosis*, % | Qualifying event | Stent type | Lesion location, N (%) | Study design |
| --- | --- | --- | --- | --- | --- | --- | --- | --- | --- | --- | --- |
| >21days | | | | | | | | | | | |
| Jiang | 2011 | 33.6 | China | 100 | 53.2 | 87.0 | ≥ 70 | Stroke 29.5% | Wingspan | ICA+MCA+VA+BA | Perspective case series |
| Yu | 2011 | 89 | China | 60 | 64.3 | 78.3 | 78.4 | TIA 26.7%  Stroke 73.3% | Wingspan | ICA+MCA+VA+BA | Perspective case-control study |
| Yu | 2012 | 91.2 | China | 57 | 64.2 | 78.9 | 72.8 | TIA 26.3%  Stroke 73.7% | Nitol | ICA+MCA+VA+BA | Perspective case series |
| Yu | 2014 | 132.9 | China | 65 | 62.9 | 74.6 | 71.8 | NA | Wingspan | MCA+BA+VA | Perspective case series |
| Li | 2015 | 23.7 | China | 433 | 57.3 | 69.1 | 82.3 | Stroke 46.9% | Wingspan | ICA+MCA+VA+BA | Perspective case series |
| Gao | 2016 | >21 | China | 100 | 56.0 | 73.0 | 82.7 | Stroke 50% | Wingspan | ICA+MCA+VA+BA | Perspective case series |
| Liu | 2016 | 30.1 | China | 97 | 58.4 | 83.5 | BA: 83.7;  VA: 84.6 | TIA 53%  Stroke 47% | Apollo, Wingspan | VA+BA | Perspective case series |
| Alexander | 2019 | 22 | US | 152 | 61.9 | 53.3 | 70 - 99 | Stroke 100% | Wingspan stents | ICA+MCA+VA+BA+PCA | Prospective case series |
| ≤21days | | | | | | | | | | | |
| Zaidat | 2015 | 12.3 | US | 58 | 61.8 | 70.7 | 70 - 99 | TIA 41.4%  Stroke 62.1% | BMS | Intracranial artery | Randomized controlled trial |
| Derdeyn | 2014 | 7 | US | 224 | 61.0 | 57.0 | 70 - 79: 48%;  80 - 89: 41%;  90 - 99: 11% | Stroke 63%  TIA 37% | Wingspan stents | ICA+MCA+BA+VA | Randomized controlled trial |
| Wang | 2016 | 19.1 | China | 58 | 55.1 | 48.3 | 84.3 | Stroke 37.9%  TIA 62.1% | Wingspan | ICA+MCA+VA+BA | Perspective case series |
| Wang | 2015 | 19.1 | China | 88 | 59.2 | 87.5 | 84.9 | TIA 61.4%  Stroke % 38.6% | Wingspan | VA | Perspective case series |
| Miao | 2015 | 21 | China | 300 | 58.3 | 76.0 | 70 - 99 | TIA 45.7%  Stroke 54.3% | BMS, Balloon Predilation Plus Self-Expanding Stenting | ICA+MCA+VA+BA | Perspective cohort study |
| Miao* | 2015 | 21 | China | 158 | 58.0 | 80.7 | 83.0 | TIA 42.4%  Minor stroke 48.7%  Stroke 8.9% | Apollo, Wingspan | ICA+MCA+VA+BA | Perspective case-control study |

Note: * Preprocedural stenosis was reported as means or ranges.

**Abbreviation:** WHO: world health organization, US: United States, BMS: balloon mounted stent, SES: self-expanding stent, DES: drug eluting stent, ICA: internal carotid artery, MCA: middle cerebral artery, VA: vertebral artery, BA: basilar artery, VBA: vertebrobasilar artery, ACA: anterior cerebral artery, PCA: posterior cerebral artery.

**Table e-4.** Detailed peri-procedural outcomes of the included studies.

| **Study ID** | **Timing(d)** | **Volume** | Death | | | **Stroke** | | | **Stroke or death** | | |
| --- | --- | --- | --- | --- | --- | --- | --- | --- | --- | --- | --- |
|  |  |  | N | Rate (95% CI) % | SE % | n | Rate (95% CI) % | SE % | n | Rate (95% CI) % | SE% |
| Jiang 2011 | 33.6 | 100 | 0 | 0.00 |  | 5 | 5.00 (1.9-10.6) | 2.20 | 5 | 5.00 (1.9-10.6) | 2.20 |
| Yu 2011 | 89 | 60 | 2 | 3.30 (0.7-10.3) | 2.30 | 3 | 5.00 (1.4-12.7) | 2.80 | 5 | 8.30 (3.3-17.3) | 3.60 |
| Yu 2012 | 91.2 | 57 | 2 | 3.50 (0.7-10.8) | 2.40 | 1 | 1.80 (0.2-7.9) | 1.70 | 3 | 5.30 (1.5-13.4) | 3.00 |
| Derdeyn 2014 | 7 | 224 | 5 | 2.20 (0.9-4.8) | 1.00 | 33 | 14.70 (10.6-19.8) | 2.40 | 33 | 14.70 (10.6-19.8) | 2.40 |
| Yu 2014 | 132.9 | 65 | 4 | 6.20 (2.1-14.0) | 3.00 | 4 | 6.20 (2.1-14.0) | 3.00 | 4 | 6.20 (2.1-14.0) | 3.00 |
| Li 2015 | 23.7 | 433 | 3 | 0.70 (0.2-1.8) | 0.40 | 27 | 6.20 (4.2-8.8) | 1.20 | 29 | 6.70 (4.6-9.3) | 1.20 |
| Miao 2015 | 21 | 300 | 0 | 0.00 |  | 8 | 2.70 (1.3-5.0) | 0.90 | 8 | 2.70 (1.3-5.0) | 0.90 |
| Miao* 2015 | 21 | 158 | 0 | 0.00 |  | 7 | 4.40 (2.0-8.5) | 1.60 | 7 | 4.40 (2.0-8.5) | 1.60 |
| Wang 2015 | 19.1 | 88 | 0 | 0.00 |  | 1 | 1.10 (0.1-5.2) | 1.10 | 1 | 1.10 (0.1-5.2) | 1.10 |
| Zaidat 2015 | 12.3 | 58 | 3 | 5.20 (1.5-13.2) | 2.90 | 14 | 24.10 (14.6-36.2) | 5.60 | 14 | 24.10 (14.6-36.2) | 5.60 |
| Liu 2016 | 30.1 | 97 | 0 | 0.00 |  | 4 | 4.10 (1.4-9.5) | 2.00 | 4 | 4.10 (1.4-9.5) | 2.00 |
| Wang 2016 | 21 | 58 | 0 | 0.00 |  | 1 | 1.70 (0.2-7.8) | 1.70 | 1 | 1.70 (0.2-7.8) | 1.70 |
| Alexander 2019 | 22 | 152 | 2 | 1.30 (0.3-4.2) | 0.90 | 2 | 1.30 (0.3-4.2) | 0.90 | 4 | 2.60 (0.9-6.1) | 1.30 |

**Table e-5.** Detailed post-procedural outcomes of the included studies.

| Study ID | Timing(d) | Volume | Death | | | Stroke | | | Stroke or death | | |
| --- | --- | --- | --- | --- | --- | --- | --- | --- | --- | --- | --- |
|  |  |  | **N** | Rate % | **SE %** | **N** | Rate % | **SE %** | **N** | Rate % | **SE%** |
| Jiang 2011 | 33.6 | 99 | 1 | 1.0 (0.1-4.6) | 1.0 | 4 | 4.0 (1.4-9.3) | 2.0 | 4 | 4.0 (1.4-9.3) | 2.0 |
| Yu 2011 | 89 | 60 | 0 | 0.0 | 0.0 | 3 | 5.0 (1.4-12.7) | 2.8 | 3 | 5.0 (1.4-12.7) | 2.8 |
| Derdeyn 2014 | 7 | 221 | 4 | 1.8 (0.6-4.2) | 1.8 | 15 | 6.8 (4.0-10.7) | 1.7 | 17 | 7.7 (4.7-11.8) | 1.8 |
| Yu 2014 | 132.9 | 65 | 0 | 0.0 | 0.0 | 0 | 0.0 |  | 0 | 0.0 |  |
| Li 2015 | 23.7 | 365 | 10 | 2.7 (1.4-4.8) | 2.7 | 24 | 6.6 (4.4-9.5) | 1.3 | 31 | 8.5(6.0-11.7) | 1.5 |
| Zaidat 2015 | 12.3 | 58 | 1 | 1.7 (0.2-7.8) | 1.7 | 6 | 10.3 (4.4-20.1) | 4.0 |  |  |  |
| Wang 2015 | 19.1 | 80 | 10 | 12.5 (6.6-21.0) | 12.5 | 8 | 10.0 (4.8-18.0) | 3.4 | 13 | 16.3 (9.4-25.5) | 4.1 |
| Wang 2016 | 21 | 36 | 1 | 2.8 (0.3-12.3) | 2.8 | 5 | 13.9 (5.5-27.8) | 5.8 | 5 | 13.9 (5.5-27.8) | 5.8 |

**Table e-6.** Detailed 1-year total outcomes of the included studies.

| Study ID | Timing(d) | Volume | Death | | | Stroke | | | Stroke or death | | |
| --- | --- | --- | --- | --- | --- | --- | --- | --- | --- | --- | --- |
|  |  |  | **N** | Rate % | **SE %** | **N** | Rate % | **SE %** | **N** | Rate % | **SE%** |
| Jiang 2011 | 33.6 | 99 | 1 | 1.0 (0.1-4.6) | 1.0 | 9 | 9.1 (4.6-15.9) | 2.9 | 9 | 9.1 (4.6-15.9) | 2.9 |
| Yu 2011 | 89 | 60 | 2 | 3.3 (0.7-10.3) | 2.3 | 6 | 10.0 (4.3-19.5) | 3.9 | 8 | 13.3 (6.5-23.6) | 4.4 |
| Derdeyn 2014 | 7 | 221 | 9 | 4.1 (2.0-7.3) | 1.3 | 48 | 21.7 (16.7-27.5) | 2.8 | 50 | 22.6 (17.5-28.5) | 2.8 |
| Yu 2014 | 132.9 | 65 | 4 | 6.2 (2.1-14.0) | 3.0 | 4 | 6.2 (2.1-14.0) | 3.0 | 4 | 6.2 (2.1-14.0) | 3.0 |
| Li 2015 | 23.7 | 365 | 13 | 3.6 (2.0-5.8) | 1.0 | 51 | 14.0 (10.7-17.8) | 1.8 | 60 | 16.4 (12.9-20.5) | 1.9 |
| Zaidat 2015 | 12.3 | 58 | 4 | 6.9 (2.4-15.6) | 3.3 | 20 | 34.5 (23.2-47.2) | 6.2 |  |  |  |
| Wang 2015 | 19.1 | 80 | 10 | 12.5 (6.6-21.0) | 3.7 | 9 | 11.3 (5.7-19.5) | 3.5 | 14 | 17.5 (10.4-26.9) | 4.2 |
| Wang 2016 | 21 | 36 | 1 | 2.8 (0.3-12.3) | 2.7 | 6 | 16.7 (7.3-31.2) | 6.2 | 6 | 16.7 (7.2-31.2) | 6.2 |

**Table e-7.** Pooled analysis and meta-regression analysis of outcomes of intracranial stenting between time interval of ≤ 21 and > 21 days.

| Outcomes | No. of studies | Total (%) (95% CI) | Early stenting (%) (95% CI) | Delayed stenting (%) (95%CI) | I^2^_T_ (%) | I^2^_E_(%) | I^2^_D_ (%) | IRR (95% CI) | P Value |
| --- | --- | --- | --- | --- | --- | --- | --- | --- | --- |
| Peri-procedural outcomes | | | | | | | | | |
| Stroke | 11 | 4.92(2.50-8.03) | 6.33(1.82-13.02) | 3.68(1.83-6.07) | 84.2 | 90.35 | 57.92 | 1.59(1.07-2.36) | **0.021** |
| Death | 11 | 0.32(0.00-0.97) | 0.42(0.00-1.94) | 0.35(0.01-0.98) | 46.68 | 69.08 | 0.00 | 1.59(0.52-4.87) | 0.414 |
| Stroke or death | 11 | 5.16(2.74-8.21) | 6.33(1.82-13.02) | 4.32(2.61-6.38) | 83.11 | 90.35 | 35.67 | 1.45(0.98-2.12) | 0.059 |
| Post-procedural outcomes | | | | | | | | | |
| Stroke | 6 | 6.98(5.09-9.13) | 8.24(5.61-11.27) | 5.91(3.89-8.29) | 14.3 | 0.00 | NA | 1.43(0.87-2.35) | 0.164 |
| Death | 6 | 2.92(1.00-5.63) | 3.92(0.45-9.79) | 2.23(1.01-3.86) | 65.02 | 75.84 | NA | 1.71(0.79-3.68) | 0.171 |
| Stroke or death | 5 | 8.76(5.62-12.46) | 11.47(5.76-18.68) | 7.35(5.11-9.95) | 56.54 | 60.69 | NA | 1.37(0.86-2.20) | 0.181 |
| 1-year total outcomes | | | | | | | | | |
| Stroke | 6 | 16.81(11.25-23.18) | 20.40(12.37-29.77) | 12.79(9.87-16.02) | 77.52 | 72.99 | NA | 1.62(1.17-2.27) | **0.004** |
| Death | 6 | 4.32(2.06-7.23) | 6.13(2.58-10.85) | 2.83(1.45-4.62) | 60.82 | 54.61 | NA | 2.01(1.04-3.89) | **0.037** |
| Stroke or death | 5 | 16.51(12.21-21.30) | 20.55(16.33-25.11) | 14.66(11.56-18.06) | 58.45 | 0.00 | NA | 1.40(1.00-1.95) | **0.049** |

NA not applicable

*CI* confidence interval, *I^2^* the variation attributable to heterogeneity, *I^2^_T_* heterogeneity in overall meta-analysis, *I^2^*_≤_ heterogeneity in the timing ≤ 21 days group, *I^2^_>_* heterogeneity in the timing > 21 days group, *IRR* incidence rate ratio, univariate meta-regression analysis was adjusted by time of intervention (timing ≤ 21days compared to timing >21 days)

**Figure e-6.** Forest plot of periprocedural death.

**Figure e-7.** Forest plot of periprocedural stroke.

**Figure e-8.** Forest plot of periprocedural stroke or death.

**Figure e-9.** Forest plot of post-procedural death.

**Figure e-10.** Forest plot of post-procedural stroke.

**Figure e-11.** Forest plot of post-procedural stroke or death.

**Figure e-12.** Forest plot of 1-year total death.

**Figure e-13.** Forest plot of 1-year total stroke.

**Figure e-14.** Forest plot of 1-year total stroke or death.


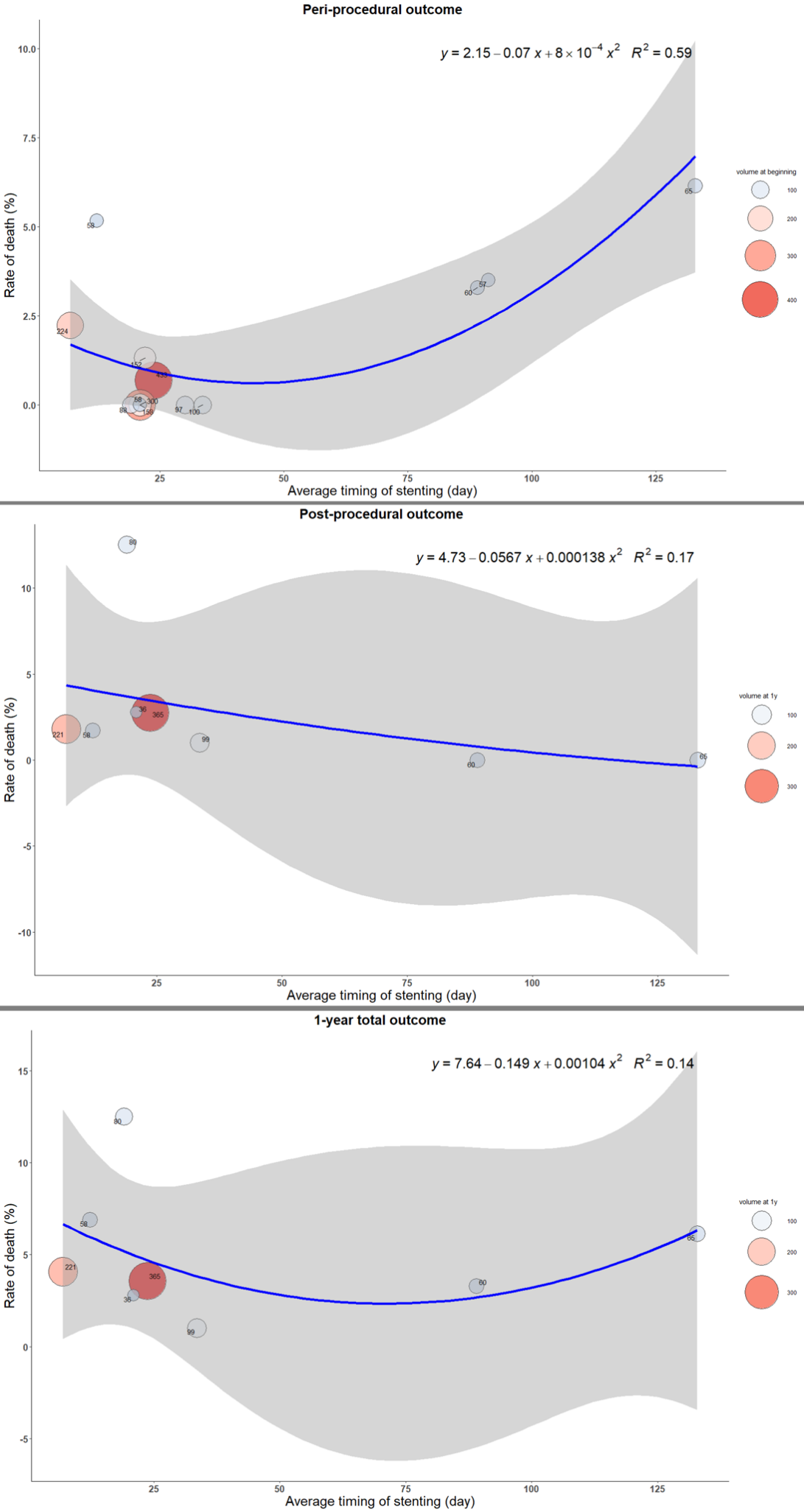


**Figure e-15.** The trend of death with timing in different stages.


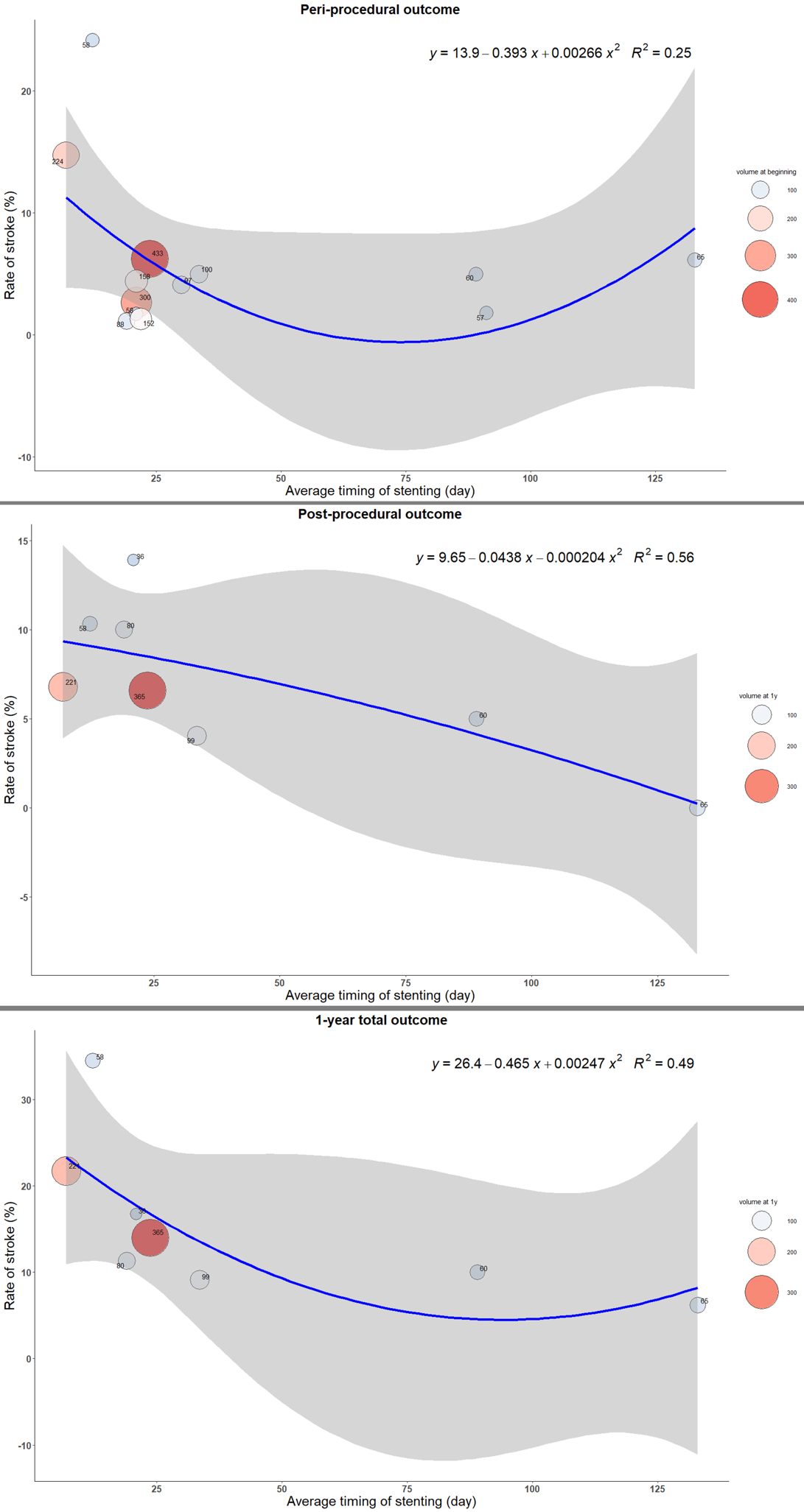


**Figure e-16** The trend of stroke with timing in different stages.


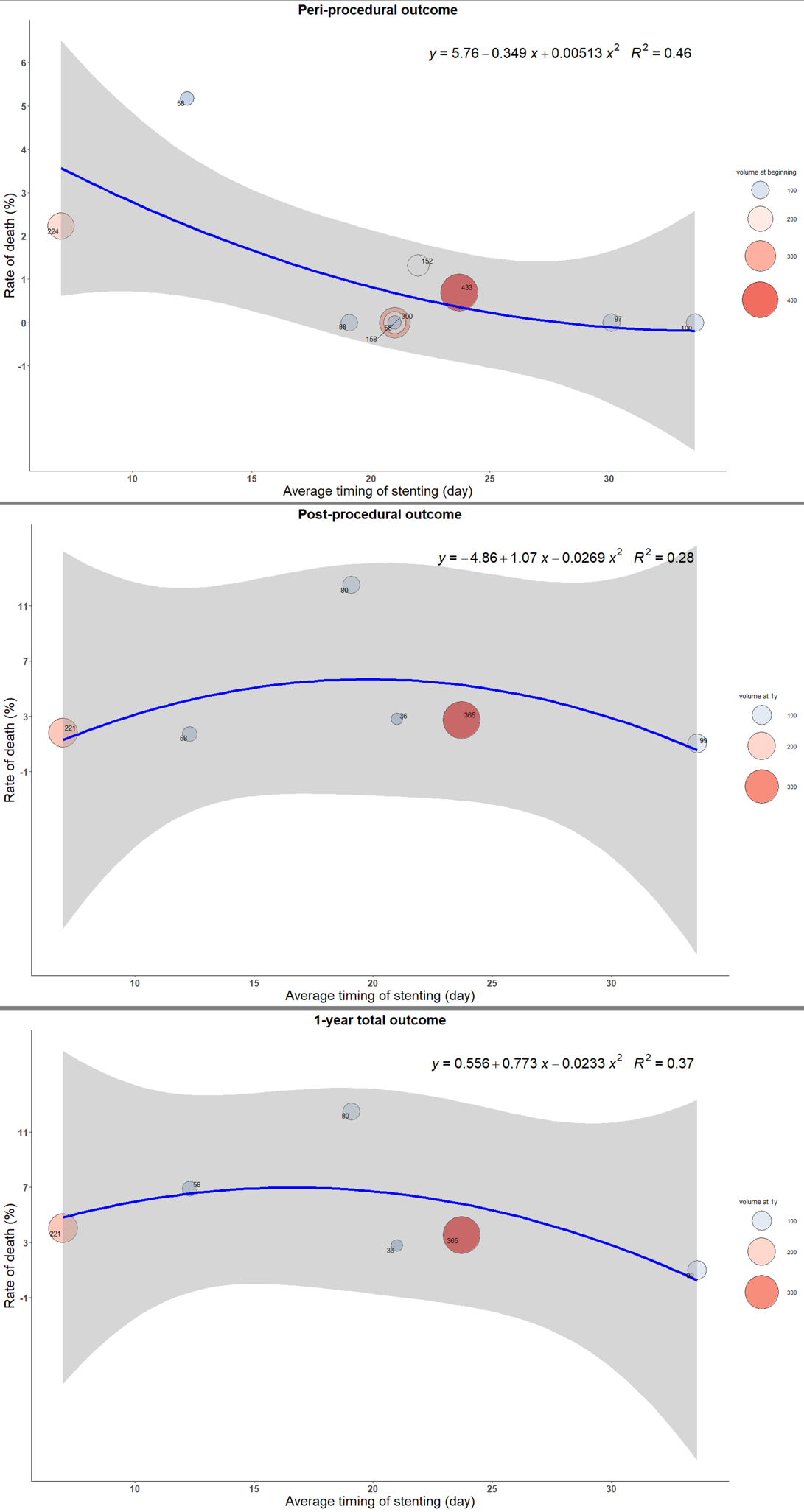


**Figure e-17.** The trend of death with timing of stenting in different stages in sensitivity analysis.


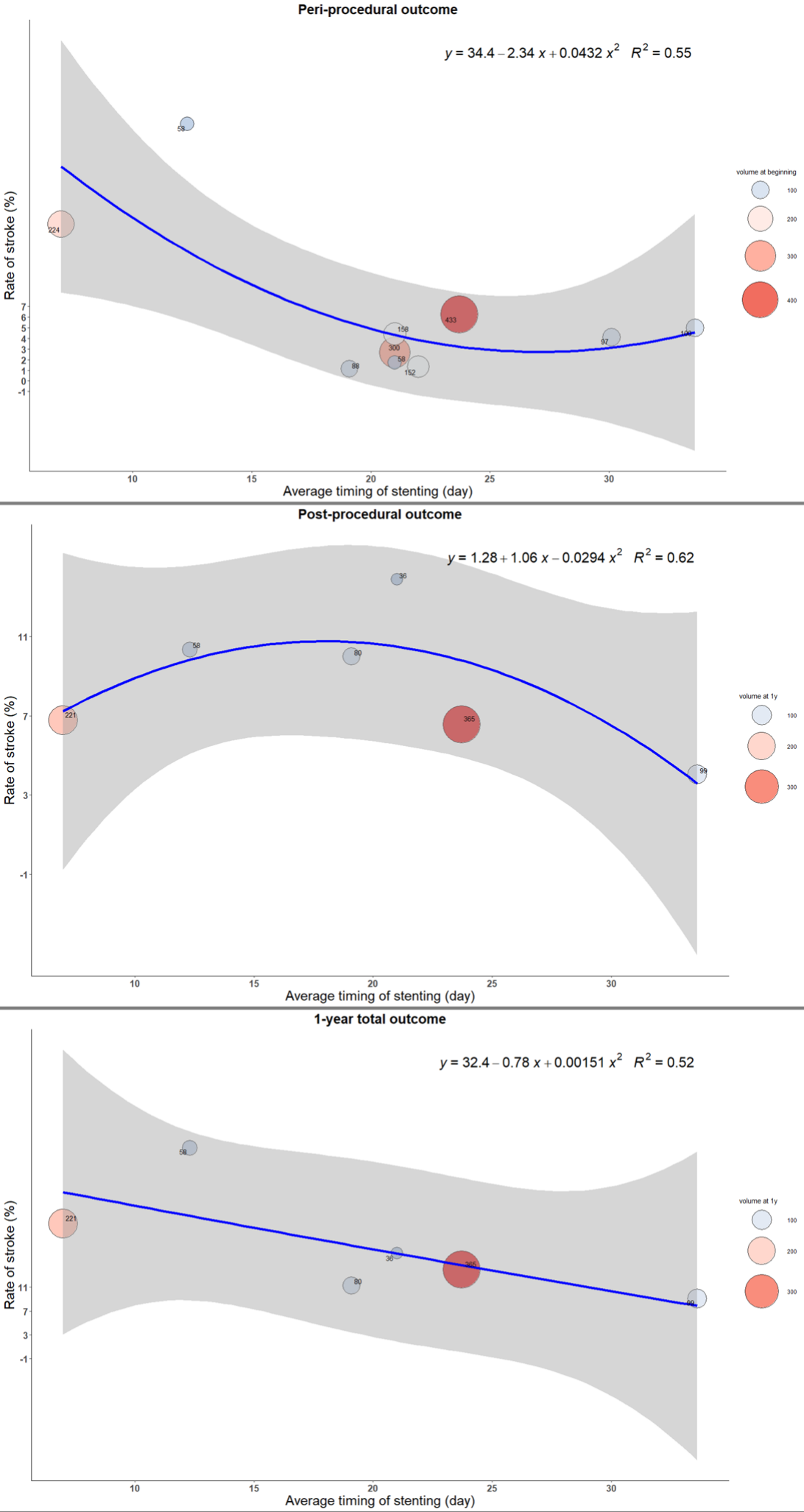


**Figure e-18.**  The trend of stroke with timing of stenting in different stages in sensitivity analysis.

**Supplementary References**

1. Jiang W J, Yu W, Du B, Gao F, Cui L Y. Outcome of patients with >/=70% symptomatic intracranial stenosis after Wingspan stenting. *Stroke*. 2011;42:1971-1975.

2. Yu S C, Leung T W, Lee K T, Hui J W, Wong L K. Angioplasty and stenting of atherosclerotic middle cerebral arteries with Wingspan: evaluation of clinical outcome, restenosis, and procedure outcome. *AJNR American journal of neuroradiology*. 2011;32:753-758.

3. Yu S C, Leung T W, Hung E H, Lee K T, Wong L K. Angioplasty and stenting for intracranial atherosclerotic stenosis with nitinol stent: factors affecting technical success and patient safety. *Neurosurgery*. 2012;70:104-113.

4. Derdeyn C P, Chimowitz M I, Lynn M J, Fiorella D, Turan T N, Janis L S, et al. Aggressive medical treatment with or without stenting in high-risk patients with intracranial artery stenosis (SAMMPRIS): the final results of a randomised trial. *Lancet (London, England)*. 2014;383:333-341.

5. Yu S C, Leung T W, Lee K T, Wong L K. Angioplasty and stenting of intracranial atherosclerosis with the Wingspan system: 1-year clinical and radiological outcome in a single Asian center. *Journal of neurointerventional surgery*. 2014;6:96-102.

6. Li T X, Gao B L, Cai D Y, Wang Z L, Zhu L F, Xue J Y, et al. Wingspan Stenting for Severe Symptomatic Intracranial Atherosclerotic Stenosis in 433 Patients Treated at a Single Medical Center. *PloS one*. 2015;10:e0139377.

7. Miao Z, Song L, Liebeskind D S, Liu L, Ma N, Wang Y, et al. Outcomes of tailored angioplasty and/or stenting for symptomatic intracranial atherosclerosis: a prospective cohort study after SAMMPRIS. *Journal of neurointerventional surgery*. 2015;7:331-335.

8. Miao Z, Zhang Y, Shuai J, Jiang C, Zhu Q, Chen K, et al. Thirty-Day Outcome of a Multicenter Registry Study of Stenting for Symptomatic Intracranial Artery Stenosis in China. *Stroke*. 2015;46:2822-2829.

9. Wang Z L, Gao B L, Li T X, Cai D Y, Zhu L F, Bai W X, et al. Symptomatic intracranial vertebral artery atherosclerotic stenosis (>/=70%) with concurrent contralateral vertebral atherosclerotic diseases in 88 patients treated with the intracranial stenting. *European journal of radiology*. 2015;84:1801-1804.

10. Zaidat O O, Fitzsimmons B F, Woodward B K, Wang Z, Killer-Oberpfalzer M, Wakhloo A, et al. Effect of a balloon-expandable intracranial stent vs medical therapy on risk of stroke in patients with symptomatic intracranial stenosis: the VISSIT randomized clinical trial. *Jama*. 2015;313:1240-1248.

11. Liu L, Zhao X, Mo D, Ma N, Gao F, Miao Z. Stenting for symptomatic intracranial vertebrobasilar artery stenosis: 30-day results in a high-volume stroke center. *Clinical neurology and neurosurgery*. 2016;143:132-138.

12. Wang Z L, Gao B L, Li T X, Cai D Y, Zhu L F, Xue J Y, et al. Severe symptomatic intracranial internal carotid artery stenosis treated with intracranial stenting: a single center study with 58 patients. *Diagnostic and interventional radiology (Ankara, Turkey)*. 2016;22:178-183.

13. Gao P, Wang D, Zhao Z, Cai Y, Li T, Shi H, et al. Multicenter Prospective Trial of Stent Placement in Patients with Symptomatic High-Grade Intracranial Stenosis. *AJNR American journal of neuroradiology*. 2016;37:1275-1280.

14. Alexander M J, Zauner A, Chaloupka J C, Baxter B, Callison R C, Gupta R, et al. WEAVE Trial: Final Results in 152 On-Label Patients. *Stroke*. 2019;50:889-894.
